# Using the multiphase optimization strategy (MOST) framework to optimize an intervention to increase COVID-19 testing for Black and Latino/Hispanic frontline essential workers: A study protocol

**DOI:** 10.1186/s12889-022-13576-0

**Published:** 2022-06-21

**Authors:** Marya Gwadz, Charles M. Cleland, Maria Lizardo, Robert L. Hawkins, Greg Bangser, Lalitha Parameswaran, Victoria Stanhope, Jennifer A. Robinson, Shristi Karim, Tierra Hollaway, Paola G. Ramirez, Prema L. Filippone, Amanda S. Ritchie, Angela Banfield, Elizabeth Silverman

**Affiliations:** 1 Intervention Innovations Team Lab (IIT-Lab), NYU Silver School of Social Work, 1 Washington Square North, New York, NY 10003 USA; 2 Center for Drug Use and HIV Research (CDUHR), NYU School of Global Public Health, 708 Broadway, New York, NY 10003 USA; 3 Division of Biostatistics, Department of Population Health at NYU Grossman School of Medicine, 180 Madison Ave, New York, NY 10016 USA; 4 Northern Manhattan Improvement Corporation (NMIC), 45 Wadsworth Avenue, New York, NY 10033 USA; 5 Department of Medicine, NYU Grossman School of Medicine, New York, NY 10016 USA; 6 Independent Consultant, New York, NY 10031 USA; 7 SUNY Research Foundation, Downstate Medical Center, 450 Clarkson Ave, Brooklyn, NY 11203 USA

**Keywords:** Multiphase optimization strategy, COVID-19, Testing, RADx-UP, Frontline workers, Essential workers, Health inequality, Racial/ethnic disparities, Community-engaged, Intervention, Factorial design

## Abstract

**Background:**

Among those at highest risk for COVID-19 exposure is the large population of frontline essential workers in occupations such food service, retail, personal care, and in-home health services, among whom Black and Latino/Hispanic persons are over-represented. For those not vaccinated and at risk for exposure to COVID-19, including frontline essential workers, regular (approximately weekly) COVID-19 testing is recommended. However, Black and Latino/Hispanic frontline essential workers in these occupations experience serious impediments to COVID-19 testing at individual/attitudinal- (e.g., lack of knowledge of guidelines), social- (e.g., social norms), and structural-levels of influence (e.g., poor access), and rates of testing for COVID-19 are insufficient.

**Methods/design:**

The proposed community-engaged study uses the multiphase optimization strategy (MOST) framework and an efficient factorial design to test four candidate behavioral intervention components informed by an integrated conceptual model that combines critical race theory, harm reduction, and self-determination theory. They are A) motivational interview counseling, B) text messaging grounded in behavioral economics, C) peer education, and D) access to testing (via navigation to an appointment vs. a self-test kit). All participants receive health education on COVID-19. The specific aims are to: identify which components contribute meaningfully to improvement in the primary outcome, COVID-19 testing confirmed with documentary evidence, with the most effective combination of components comprising an “optimized” intervention that strategically balances effectiveness against affordability, scalability, and efficiency (Aim 1); identify mediators and moderators of the effects of components (Aim 2); and use a mixed-methods approach to explore relationships among COVID-19 testing and vaccination (Aim 3). Participants will be *N* = 448 Black and Latino/Hispanic frontline essential workers not tested for COVID-19 in the past six months and not fully vaccinated for COVID-19, randomly assigned to one of 16 intervention conditions, and assessed at 6- and 12-weeks post-baseline. Last, *N* = 50 participants will engage in qualitative in-depth interviews.

**Discussion:**

This optimization trial is designed to yield an effective, affordable, and efficient behavioral intervention that can be rapidly scaled in community settings. Further, it will advance the literature on intervention approaches for social inequities such as those evident in the COVID-19 pandemic.

**Trial registration:**

ClinicalTrials.gov: NCT05139927; Registered on 11/29/2021. Protocol version 1.0. May 2, 2022, Version 1.0

**Supplementary Information:**

The online version contains supplementary material available at 10.1186/s12889-022-13576-0.

## Background

### Background and rationale

Racial/ethnic disparities in COVID-19 incidence, morbidity, and mortality rates have been marked since the earliest days of the pandemic in the United States [1]. Estimated death rates from COVID-19 for Black populations are 178/100 k and 154/100 k for Latino/Hispanic populations, compared to 124/100 k among White populations and 95/100 k among Asian populations [2], attributed to structural racism and social determinants of health inequities [3–5]. Testing for COVID-19 is an essential component of the national strategy to control COVID-19, including for those who are not fully vaccinated [6]. Yet, rates of testing have dropped dramatically, which will certainly hamper efforts to control COVID-19 spread [7].

The proposed two-year study seeks to develop a community-engaged intervention to support COVID-19 testing in underserved and vulnerable populations. From the earliest days of the pandemic, social inequities between groups of workers, along with disparities in COVID-19 incidence rates, have been striking [8]. We focus on a subpopulation of the more than 30 million workers in the United States who are placed at very high risk for exposure to COVID-19 because they must physically report to their jobs and cannot work at home, called frontline essential workers (FEW) [9, 10]. Although there is no single definition of FEW, the Department of Homeland Security and the American Community Survey provide guidance on the occupational categories most likely to be FEW [11]. Among those at highest risk for exposure to COVID-19 but with the fewest protections is the large population of FEW in “lower status occupations” where vaccination is not generally required and testing not routinely provided: food preparation and serving, retail and sales, building and grounds cleaning and maintenance, personal care and service, and in-home health care [11–14]. Moreover, among FEW in these occupations, Black and Latino/Hispanic (BLH) persons are substantially over-represented, and the majority of these BLH-FEW reside or work in geographical areas with high rates of socioeconomic disadvantage, which limits access to COVID-19 testing and other vital health-protective resources [1, 12–15]. Although precise data on testing by occupation are limited, current estimates show that rates of COVID-19 testing among BLH populations are typically lower than among White populations [16, 17]. Further, only an estimated 25–50% of BLH-FEW are currently fully vaccinated [17, 18]. The proposed study, therefore, focuses on BLH-FEW in these lower status occupations.

The Centers for Disease Control and Prevention (CDC) recommend viral testing for SARS-CoV-2, the virus that causes COVID-19 (antigen and/or nucleic acid amplification [NAATs] tests), in the following circumstances. For those who show symptoms of COVID-19 (e.g., fever, chills, cough, shortness of breath, fatigue, muscle aches, headache, and loss of taste or smell), immediate diagnostic testing is recommended, along with a period of isolation, whether the individual is fully vaccinated or not [19]. Testing is recommended in additional circumstances for those not vaccinated. Diagnostic testing and re-testing are recommended after exposure to someone with a confirmed or suspected case of COVID-19 [19] and those diagnosed should remain in isolation until they meet local criteria for discontinuing isolation [19]. Further, testing asymptomatic persons without recent known or suspected exposure to COVID-19, but who are at risk for exposure, is critical for early identification, isolation, and disease prevention [19]**.** Indeed, persons with asymptomatic or pre-symptomatic infection are frequent contributors to community SARS-CoV-2 transmission. Among those groups the CDC recommends prioritizing for this regular screening testing include racial and ethnic minority groups and other populations disproportionately affected by COVID-19 and workers in high-density worksites or worksites with large numbers of close contacts to co-workers or customers (e.g., restaurant workers, grocery store workers) [19]. The proposed study, therefore, adheres to CDC guidance on priority populations, scenarios for SARS-CoV-2 testing and mitigation strategies, and also aligns with local guidelines. Currently, the CDC and our local health department in New York City recommend testing approximately weekly for those who are not fully vaccinated but who may be potentially exposed to COVID-19, such as FEW in lower status occupations [20, 21].

The proposed study is led by a collaborative team at New York University (NYU) and the Northern Manhattan Improvement Corporation (NMIC). NYU is a large research university. NMIC is a large and well-established community-based organization founded in 1979 that serves the diverse needs of BLH in vulnerable and under-served communities. The proposed study builds on NMIC’s experience with BLH-FEW, deep understanding of barriers to COVID-19 testing among BLH-FEW, and expertise in implementing health promotion interventions in community settings. The research team at NYU is highly experienced in designing and testing culturally salient behavioral interventions with BLH populations that address barriers similar to those that impede COVID-19 testing [22–24].

The study is guided by a framework called the Intervention Innovations Team integrated conceptual model (IIT-ICM) that was developed by our research team to guide the creation and refinement of culturally and structurally salient behavioral interventions to address health inequity in the United States. The IIT-ICM combines critical race theory, harm reduction, and self-determination theory [25, 26]. In the IIT-ICM, we “center” the experiences of BLH-FEW, focus on racism and discrimination (not just race/ethnicity) and contextual barriers, attend to counter-narratives, and highlight strengths and resilience [27]. Further, we attend to barriers to/facilitators of health inequities (such as low rates of COVID-19 testing for BLH-FEW populations in this case) at multiple levels of influence [28], namely, at individual, social, and structural levels [29], similar to other racial/ethnic health disparities [5]. The IIT-ICM highlights that these multi-level influences are shaped by the larger culture, primary among them structural racism, discrimination, and past and present maltreatment of BLH populations by institutions and systems [30]. Further, the IIT-ICM underscores the value of any positive change, and the importance of autonomy supportive approaches. As we describe in more detail below, the motivational interviewing (MI) counseling approach naturally aligns with the IIT-ICM. In the sections that follow we describe the barriers that BLH-FEW experience to testing for COVID-19, organized by individual/attitudinal-, social-, and structural-level influences [29].

Individual/attitudinal-level barriers to COVID-19 testing include insufficient knowledge about testing guidelines, and health beliefs and emotions such as low perceived risk for and low perceived severity of COVID (leading to COVID-19 being experienced as a distant threat), fear of consequences of a positive test (unemployment, eviction, deportation), distrust of institutional sources of information, and counter-narratives/conspiracy theories about COVID-19 and testing [31–35]. Further, there is growing interest in how cognitive biases and heuristics impede behavioral intentions to carry out a health behavior such as COVID-19 testing [36]. Indeed, individuals typically show evidence of biases in judgment and reliance on heuristic ‘‘shortcuts’’ for health decisions [37, 38], such as present-bias (the tendency to meet current desires or needs at the price of future beneficial outcomes) and information salience (acting on the information that first comes to mind rather than on all the relevant information available) [39–41]. At the social level of influence, social norms impede regular COVID-19 testing (e.g., norms that support delaying or declining COVID-19 testing) [31, 42]. At the same time, altruism and a sense of collective responsibility can be harnessed to support testing and tap into community resilience [31, 42]. Structural-level barriers are systemic issues that impact one's ability to access a needed service [43–45]. Structural barriers impede access to testing. These include insufficient local testing sites, language barriers, and lack of paid sick leave [46, 47].

The multiphase optimization strategy (MOST) framework. There is growing awareness regarding the need for rigorous research designs including the multiphase optimization strategy (MOST) [48]. The proposed study leverages the MOST framework to advance interventions for the challenge of insufficient COVID-19 testing uptake among BLH-FEW. The objective of MOST is to improve and strategically balance intervention effectiveness, affordability, scalability, and efficiency (“EASE”) using a three phase-model (preparation, optimization, and evaluation), and designs such as factorial experiments. The preparation phase entails identifying promising candidate intervention components and developing a conceptual model, and the optimization phase comprises the systematic testing of the candidate intervention components, the most promising of which, based on pre-specified criteria (called the “optimization objective”), can then be combined into a multi-component intervention [48]. This optimized intervention can then be tested in a randomized controlled trial ([RCT], i.e., the evaluation phase). MOST is economical because multiple intervention components can be tested simultaneously. By testing effects of *individual intervention components* and their interactions, the MOST framework can determine which candidate components contribute to effectiveness, how the presence of one component affects the performance of another, and which components can be eliminated to avoid including lengthy and costly components with little benefit. The candidate intervention components are adaptations of existing acceptable, feasible, and effective intervention approaches. Informed by the IIT-ICM, the candidate behavioral intervention components to be tested in the present study are: A) motivational interview (MI) counseling, B) a text message (TM) intervention grounded in behavioral economics (BE), C) peer education, and D) access to COVID-19 testing (via navigation or a self-test kit).

The optimization objective. In MOST, the optimization objective is the criteria used to guide the decision making to create the new optimized intervention from the separate candidate components. The proposed study’s optimization objective is to create an efficient multi-component intervention from the candidate components with no inactive, poorly performing, or counter-productive elements. For example, depending on findings, the optimized intervention may be comprised of one or two of the most effective components and a core session (the standard of care). This new efficient multi-component intervention can then be rapidly implemented at NMIC and other community-based organizations for maximum public health benefit, and tested in future research. We have completed the preparation phase for the proposed study, as we describe in the next section. The proposed study seeks to carry out the optimization phase; namely, an efficient factorial experiment to test four candidate intervention components and from the most effective of these, optimize a multicomponent intervention. To date, NIH has funded more than 100 studies using the MOST framework and our research team is highly experienced with MOST [24, 49].

The overall goal of the preparation phase in MOST is to create a conceptual model and identify and refine promising candidate components to address theoretical mediators in the model (that is, candidate components that show acceptability, feasibility, and evidence of effectiveness). In the planning phases of this study, in collaboration with a Community Advisory Board (CAB) comprised of BLH individuals, including BLH-FEW, we explored the utility of the MOST framework for the problem of insufficient COVID-19 testing among BLH-FEW. CAB members are BLH including clients at NMIC and FEW and diverse with respect to age, sex, and occupation. They are highly knowledgeable about barriers to COVID-19 testing in their communities, and potential solutions. The CAB will have an active role in all study phases.

**Fig. 1:**
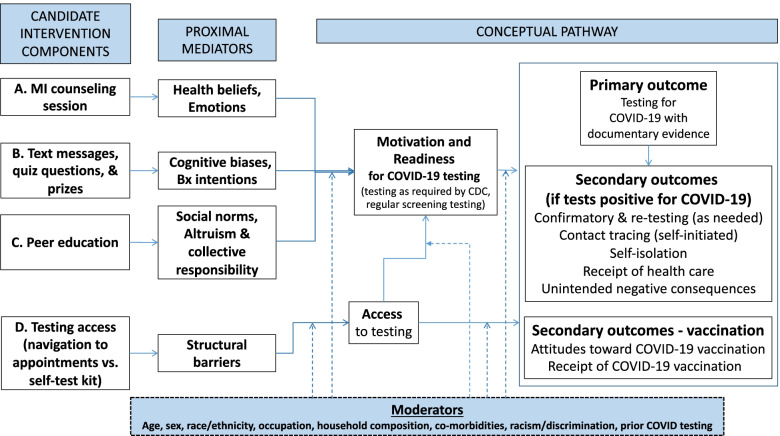
Conceptual Model Grounded in the Intervention Innovations Team Integrated Conceptual Model (IIT-ICM)

We used the ADAPT-ITT model [50], a well-established framework for adapting evidence-based interventions to new populations, to guide the process of developing the conceptual model and identifying candidate intervention components for this problem. First, we assessed risk in the new population from the perspectives of CAB members and reviewed the literature on barriers to COVID-19 testing and potential solutions. We created a comprehensive conceptual framework grounded in the IIT-ICM that described multi-level barriers to COVID-19 testing (Fig. 1). We focused on important but modifiable barriers to COVID-19 testing. We grouped individual/attitudinal-level barriers into health beliefs and emotions (e.g., low perceived risk, distrust, fear) and cognitive biases and behavioral intentions. Insufficient knowledge was another important individual-level barrier. Social-level barriers focused on social norms that impede testing and factors that facilitate COVID-19 testing (altruism and collective responsibility), and structural-level barriers are those that impede access to testing. Next, we focused on how best to address these barriers, selecting clinical approaches and modalities of behavior change that align with the IIT-ICM. With the CAB, we reviewed promising intervention approaches for each type of barrier, focusing mainly on our own past effective interventions with BLH populations. We prioritized candidate components that were brief or that would require only minimal staff time, to support future scale-up of the optimized intervention. All components are culturally salient in that they reflect the specific barriers to COVID-19 testing experienced by BLH-FEW. In an iterative process, we selected the following candidate behavioral intervention components: A) motivational interview (MI) counseling, B) a text message (TM) intervention grounded in behavioral economics (BE), C) peer education, and D) access to testing (level 1: navigation to testing appointments vs. level 2: provision of a self-test kit; we explain why Component D contrasts alternative strategies in Approach). Further, we determined all participants would receive a core intervention comprised of the standard of care; namely, health education on COVID-19 testing and referrals to testing sites that provide FDA-authorized or approved COVID-19 testing, in compliance with CDC and local guidelines [19]. Third, we worked with the CAB to create the content for components (e.g., core messages for peer education in Component C, TMs), refined in an iterative fashion. In step 4 of the ADAPT-ITT process, we developed manuals for a core intervention session and the four candidate components while maintaining fidelity to the core elements, behavioral theory, and internal logic of the original evidence-based interventions. In step 5, the CAB reviewed the first drafts of candidate components, and feedback was incorporated (step 6). In a final step, we used qualitative cognitive interviewing [51] with CAB members to “walk through” candidate components; then the feedback was incorporated. The study’s primary outcome is COVID-19 testing and secondary outcomes were identified (Fig. 1), including COVID-19 vaccination. Refinement of components will continue in the proposed study. Next, we briefly describe the evidence base for candidate components.

Addressing health beliefs and emotions using the MI counseling approach. MI is an evidence-based directive and collaborative approach for behavior change that elicits participants’ values, perspectives, and questions, identifies ambivalence and discrepancies, and corrects misinformation with permission, to thereby foster durable intrinsic motivation and readiness for change [52]. In reviews and metanalyses, MI interventions, including single-session MI interventions [53], have been found effective at clinically significant levels for a range of health behaviors [54–56]. MI has been found to be particularly effective with BLH populations compared to White populations [57, 58]. As a non-coercive, strengths-based, and autonomy-supportive approach, it may have utility in particular when health beliefs and emotions such as distrust/fear impede behavior change [59–62]. Because MI does not rely on persuasion and is non-didactic, it has been recommended by the CDC for the problem of COVID-19 testing and contact tracing [63]. We have used MI in numerous studies [22, 24, 60, 61], including an RCT for BLH persons living with HIV who had delayed or declined HIV medication. Indeed, many barriers to HIV medication are similar to those that impede COVID-19 access (e.g., distrust, fear) [64, 65]. The HIV intervention was feasible and acceptable and the odds of undetectable HIV viral load (the primary outcome) were more than five times greater in the intervention condition (OR = 5.20) [60]. Component A will be comprised of a single counseling session using evidence-based MI techniques, as we describe in the Approach.

Circumventing cognitive biases/heuristics and changing behavioral intentions using BE. BE is a systematic framework to investigate human actions [66]. BE is grounded in traditional economics in that both perspectives accept the premise that people make decisions based on costs and benefits [39, 67]. BE enriches traditional economics with insights from psychology [39, 66, 67]. BE recognizes that people have limited cognitive capacity and may feel overwhelmed when carrying out a complex task [39, 66, 67]. Recent work grounded in BE uses incentive-based strategies, including lottery-type prizes, to motivate health behavior, while considering types of biases that impede such behavior (e.g., present bias) [68]. One major advantage to BE interventions is that they can address biases and improve behavior with minimal cognitive effort [69]. Further, BE interventions typically require less staff time than traditional counseling interventions [70]. Grounded in work by Linnemayr [39], we have a program of research that uses BE approaches to overcome cognitive biases and change behavioral intentions through weekly TMs, quiz questions (QQs), and prizes to “nudge” participants toward the health behavior, HIV medication adherence [71]. Component B adapts this BE approach for the proposed study.

Changing social norms that impede testing and harnessing altruism and collective responsibility using peer education. Peer-based interventions have demonstrated high rates of acceptability and effectiveness across a range of health outcomes [72–74]. Our own research uses the peer-driven intervention (PDI) model created by Broadhead and Heckathorn [75, 76]. In PDI, participants educate peers in their social networks on concise culturally specific core messages in support of a specific health behavior change. PDI is effective because messages delivered by peers typically have more credibility than from professionals [75]. Further, when individuals educate the peers in their network about the benefits of a certain health behavior, their *own* commitment to the desired behavior is strengthened in large part because the act of educating or urging peers is a public affirmation of the desired behavior. Through peer education, social norms in a network may be altered [77]. Moreover, providing participants the opportunity to deliver education to peers increases individuals’ self-efficacy and mastery of intervention content [75]. We used the PDI approach in a study addressing low rates of enrollment of BLH persons living with HIV in AIDS clinical trials. Barriers to AIDS clinical trials include those that impede COVID-19 access (e.g., social norms) [78]. PDI condition participants were 30 times more likely to be screened for trials than controls (49.3% vs. 3.7%; *p* < 0.001) [61]. In addition to the effects of the PDI as a whole, the experience of educating peers also increased odds screening for trials (AOR = 1.4; *p* < 0.05) [61]. Component C adapts this approach and is comprised of training study participants to educate three peers who are also BLH-FEW on core messages.

Circumventing structural barriers that impede access. Navigation is an individualized efficacious intervention approach first designed to reduce disparities in cancer care for low-income women of color [79–81]. Navigators help identify and resolve barriers that individuals encounter to accessing a needed health service such as COVID-19 testing [82]. We have found the navigation approach to have utility in past studies, including because it is flexible and needs-based. Component D will address access barriers and have two “levels:” navigation vs. receipt of a COVID-19 self-test kit (Flowflex Covid-19 Rapid Antigen Home Test).

No significant harms have been identified for these behavioral interventions.

### Objectives

The study’s specific aims are to:

**Aim 1**. Identify which of four candidate behavioral intervention components contribute meaningfully to improvement in the primary outcome, COVID-19 testing with documentary evidence, and from these results, optimize an efficient multicomponent intervention**.** Participants will be English and Spanish-speaking BLH-FEW (ages 18–70 years; *N* = 448) in New York City who have not been tested for COVID-19 in the past six months and who are not fully vaccinated for COVID-19. Participants will be randomly assigned to an intervention condition, engage in the assigned components, and assessed at 6- and 12-weeks post-baseline (BL).

**Aim 2**. Identify mediators (e.g., distrust, altruism, access) and moderators (e.g., sociodemographic characteristics) of the effects of each candidate intervention component to better understand the components’ mechanisms of action and conditions under which they are most effective to advance future research and inform implementation of the optimized intervention.

**Aim 3**. Explore the relationships among barriers to, facilitators of, and uptake of COVID-19 testing and COVID-19 vaccination. In qualitative research consistent with a concurrent parallel mixed-methods design [83], we will explore participants’ experiences with and perspectives on the candidate intervention components and on COVID-19 testing and vaccination (*N* = 50) and integrate qualitative and quantitative results using the joint display method to inform intervention implementation and future research.

Further, in collaboration with the CAB, we will uncover and describe factors that may promote or impede implementation of the new optimized multi-component intervention by NMIC and other community-based settings in a timely fashion at the conclusion of the proposed study.

## Methods/design

### Trial design

The study is grounded in the MOST framework. We will test four culturally salient candidate intervention components grounded in our past research, each of which addresses a critical theoretical barrier to COVID-19 testing, and which are either brief or require only minimal staff time to implement. Informed by the IIT-ICM, the candidate components are: A) motivational interview (MI) counseling, B) a text message (TM) intervention grounded in behavioral economics (BE), C) peer education, and D) access to COVID-19 testing (via navigation or a self-test kit). All participants will also receive the standard of care, a health education information session on COVID-19 testing, along with referrals to testing sites. The candidate components will be tested using a highly efficient factorial experimental design. Participants will be randomly assigned to one of 16 intervention conditions. Follow-up (FU) assessments will be carried out at 6- and 12-weeks post-BL. Then**,** we will use pre-specified criteria to identify the most effective combination of candidate components, and this combination is the new multicomponent optimized intervention.

**Fig. 2:**
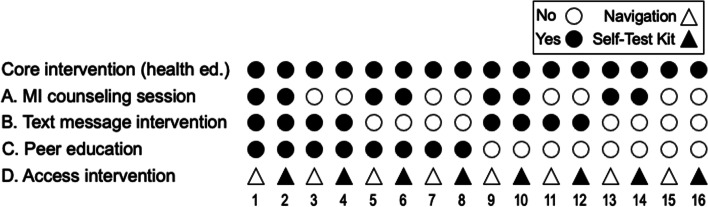
Intervention conditions in the factorial design

Each component has two levels: assigned/on vs. not assigned/off (Components A-C), or navigation vs. self-test kit (Component D). A factorial experiment testing four intervention components, each with two levels, is comprised of 16 conditions (2^4^, Fig. 2). Importantly, the design is not a 16-arm RCT. Factorial experiments separate component effects, enabling estimation of the main effect contribution of each component. Factorial experiments can be economical compared to alternative designs, because they require substantially fewer participants to achieve the same goals [48]. For example, conducting four individual experiments using the RCT design, one for each component, would require *N* = 1792 (448 participants per trial). Thus, the purpose and logical underpinnings of the factorial experiment are different from those of an RCT. The purpose of an RCT is a direct comparison of the efficacy of two or more versions of an intervention. By contrast, a factorial design never calls for a direct comparison of experimental conditions to see which one is best. Instead, the purpose is to identify which components show effectiveness. Efficiency comes from basing all estimated main effects on all 16 conditions in the factorial experiment. For example, the main effect of Component C will be estimated by comparing the mean outcome across Conditions 1–8 vs. across Conditions 9–16. All participants are included in the estimate of each main effect. Factorial experiments can have a small per-condition *N* (*N* = 28) and still achieve study aims if the total *N* (*N* = 448) is sufficient. All participants receive the core intervention, two conditions (15 & 16) receive 1 component (access, either navigation or a self-test kit), 2 conditions (1 & 2) receive all components, and the remaining 12 conditions receive 2–3 components (Fig. 2).

## Methods

### Methods: Participants, interventions, and outcomes

#### Study setting

The study will recruit BLH-FEW from the local community. The study activities will be carried out at a New York University research field site in lower Manhattan, which is centrally and conveniently located and accessible by numerous subway and bus lines. BLH-FEW can participate there in-person or virtually.

### Eligibility criteria

We focus on the large subset of lower status FEW occupations that are common in urban settings; where risk for exposure to COVID-19 is high because of frequent close contact with others in indoor settings by nature of the occupations; where COVID-19 testing and vaccination are typically not mandated for employment (in contrast to some health care and educational settings, for example); where on-site COVID-19 testing programs are very rare, and where BLH are substantially over-represented [11–14].

The study eligibility criteria are: 1) age 18–70 years; 2) can engage in study activities in English or Spanish; 3) Black or African American (including Caribbean, African, or multi-ethnic Black) and/or Latino or Hispanic race/ethnicity; 4) resides in NYC; 5) in the past month, was employed as a frontline worker in a lower status essential occupation in one or more of these domains: food preparation and serving (e.g., deli, bodega, restaurants, fast food), retail and sales (e.g., grocery, drug, and convenience stores), building and grounds cleaning and maintenance, personal care and service (e.g., in-home childcare workers, barbers, nail technicians, cosmetologists), and in-home health care services (e.g., home health aides) [11–15]; 6) has a phone that can be used for study participation and can receive TMs; 7) is not fully vaccinated for COVID-19; 8) has not been tested for COVID-19 in the past six months; 9) if previously diagnosed with COVID-19, has not been symptomatic in the past two weeks or 90 days has passed since treatment with monoclonal antibodies or convalescent plasma [84]; 10) has not been educated/interviewed as a peer for Component C; 11) willing to engage in a core session and be randomly assigned to receive 1–4 intervention components; and 12) not a member of the CAB.

The interventions will be carried out by trained and experienced behavioral interventionists with a master’s degree or higher in fields such as social work, psychology, or public health.

### Interventions

Each candidate intervention component is guided by a manual in English and Spanish that includes handouts. Manuals will be comprised of a series of exercises and will be constructed to be interactive and engaging. The candidate components, grounded in the IIT-ICM, are culturally and structurally salient and take a strengths-based and autonomy-supportive approach. Health information will be drawn from the CDC and will be reviewed for medical accuracy by a medical expert who is a study Co-Investigator. Components address different theoretical mediators and are designed to be distinct from each other. For example, the TMs are informational health messages and intended to maintain interest and engagement while the core messages for Component C are designed to tap into norms, altruism, and collective responsibility. The behavior change process for each component (e.g., MI, BE, peer education, or access) is grounded in and/or compatible with the IIT-ICM.

Core session: Standard of care (health education on COVID-19 testing). All participants receive a health education session (20–40 min) which comprises the standard of care. The goal is to increase knowledge regarding current COVID-19 testing guidelines, including for BLH-FEW, types of tests, prevention and mitigation recommendations, and provide referrals to sites that provide FDA-authorized or approved COVID-19 testing, and (optional), referral to no-cost COVID-19 vaccination site in addition to testing sites [85]. Because all participants receive the core session, its effects on the primary outcome are not assessed. It will be included in the optimized intervention.

Component A. MI counseling. Those assigned to receive Component A will engage in a MI single session lasting approximately 30–45 min. The overall goal of the session is to increase participants’ motivation and readiness to test for COVID-19 in various circumstances. The session uses the Engage, Focus, Evoke, and Plan framework and draws on evidence-based MI techniques such as highlighting change talk and identifying discrepancy [52] and supporting participant autonomy and personal decisions about COVID-19 testing. Uncovering and discussing the structural causes of health disparities such as structural racism and freely discussing fears and counter-narratives helps build motivation and readiness. The primary core elements of this component include evoking views on COVID-19 and testing (including its severity, skepticism, counter-narratives, distrust, fears) and why we have medical and institutional distrust and counter-narratives (historical factors and structural racism); with permission and using the Elicit-Provide-Elicit method, addressing perceived COVID-19 severity and the importance of testing by providing information and data on BLH-FEW using graphics and maps; rating readiness for COVID-19 testing in various scenarios (when symptomatic, when exposed, serial screening testing) on a 1–10 scale and discuss; highlighting ambivalence and/or identify discrepancy between values and behavior to build motivation (as appropriate); and planning for testing. Theoretical targets: Health beliefs (perceived risk and severity, distrust of institutions, counter-narratives), emotions (fear of consequences if diagnosed including related to immigration status).

Component B. Text messages (TMs) and quiz questions (QQs; 6 weeks). This component is grounded in principles of BE. Its main goal is to add interest and excitement to the goal of COVID-19 testing, serve as a reminder that COVID-19 testing is recommended in a number of scenarios, and “nudge” participants toward testing and creating a habit of serial screening when asymptomatic and COVID-19 testing as needed. Participants first receive a brief orientation to the component (15 min.), and the participant will put the study phone number into his/her phone and a test TM and QQ will be sent. TMs and QQs are programmed into the Twilio program and sent automatically. Twice a week participants will receive a TM with information about COVID-19 and COVID-19 testing followed by a true/false question about that TM two days later, for which they earn 10 points for a correct answer, and 5 points for an incorrect answer. Those who provide the correct answer receive a message of congratulations and those who answer incorrectly receive a TM with the correct answer. Participants earn modest prizes based on their points. Participants receive feedback by TM on the number of points received to date and reminders that they have the chance to win prizes. At the 3rd week, those with high points (30–60 points) receive a prize of $25 and the remainder receive $10. At the 6th week, those with high points (60–120) receive $25 and the remainder receive $10. Participants also have a chance to win a lottery prize if tested for COVID-19 and documentary evidence is provided by the 6-week FU assessment (if tested, 3/10 chance of winning $50 and 7/10 chance of $25, if not tested or documentary evidence is not provided, $15 participation bonus is provided). The prize amount is determined by spinning a prize wheel. TMs are comprised of information about COVID-19 and testing from the CDC website. Examples of TMs include: We know some people are waiting to get vaccinated. Health officials recommend regular (weekly) COVID-19 testing for people who are not fully vaccinated, and COVID-19 testing is quick, easy, and available for free at hundreds of locations without an appointment. The TMs are intended to be informational, but not necessarily to tap into altruism and social norms, which are the focus of Component C. Theoretical targets: cognitive biases, behavioral intentions.

Component C. Peer education. This component has two aspects: Participants are trained to educate their peers on a set of core messages about the importance of COVID-19 testing that address social norms about COVID-19 testing and highlight COVID-19 testing as an altruistic act (15–20 min. training). Then, participants are given the opportunity to educate three peers in their existing social networks on the core messages, including other BLH-FEW. These peers contact the study directly and receive a brief assessment and referrals to testing, but are not enrolled in the study. The core messages (Table 1) were developed with our CAB and the peer education procedures have been tested in numerous studies [61, 75, 76]. The component manual includes the following activities: **a)** Participants receive an overview of the component and a review and discussion of the core messages. **b)** Then, we review when and how to educate peers; namely: whom to approach (individuals who they know by name or face and have seen in the past 30 days who are aged 18 and older, and who are not already enrolled in the study), when to approach (at a time that mutually convenient and that does not disrupt work), where peer education should take place (in a confidential and private location), how to avoid COVID-19 risk during peer education (adhere to public health prevention guidelines such as masking and social distancing or conduct peer education virtually), and how to conduct the education (introduce the core messages using an IRB-approved script, discuss but do not coerce testing). **c)** Participants will receive materials such as the script, a wallet card with the core messages (to guide peer education), and three coded coupons that the peer can use to contact the study directly to complete the brief interview. **d)** Participants will be provided with referrals to COVID-19 testing sites and COVID-19 vaccination in the event peers wish to discuss testing/vaccination. **e)** Participants are encouraged to conduct the peer education in the next 3 weeks; coupon numbers expire after that period of time. Peers contact the study directly, receive a brief assessment including age, race/ethnicity, sex, occupation, and true/false questions on the core messages, and are offered referrals to COVID-19 testing and vaccination. These procedures are intended to motivate a thorough peer education experience by the peer and document peer education. Participants receive compensation ($15) when the peer contacts the study and a bonus of $10 if the peer answers ≥ 50% of the true/false questions correctly. This compensation approach is designed to motivate participants to carry out the peer education. The peer also receives compensation for the brief interview ($25). Peers are not eligible for enrollment into the factorial experiment. Participants can educate peers even if they have not been tested for COVID-19 or made their final decisions about testing. Core messages will be reviewed regularly for medical accuracy and changed or edited as needed. Theoretical targets: social norms, altruism and collective responsibility.

**Table 1:** Core messages used in peer education for Component C

1. Regular COVID testing is still an important part of fighting the COVID-19 epidemic in our community
2. Weekly COVID testing is recommended for people who are not fully vaccinated yet
3. COVID testing is quick, easy, and available for free at hundreds of locations without an appointment
4. Many people don’t know they have COVID-19. Getting tested regularly is one important tool to protect yourself and your community
5. Regular testing matters more for groups affected the most by COVID-19, such as Black and Latino people and frontline and essential workers
6. Many New Yorkers aren’t fully vaccinated for COVID yet, so getting tested regularly for COVID is critical for stopping the spread of COVID in our city
7. There are different ways to get tested for COVID, including self-tests that can be used at home, and testing at a health care facility
8. Millions of Black and Latino essential and frontline workers are doing their part and getting tested for COVID-19
9. If you think you’ve been exposed to the COVID virus, you can do your part by getting a COVID test as soon as you can
10. If you test positive for COVID, inform your health care provider or call 311 to find out what to do to keep yourself and your community safe

Component D. Access (Level 1: Navigation to FDA-authorized or approved COVID testing, Level 2: self-test kit). Level 1: Navigation is comprised of a single brief session (20–30 min) that includes guidance to assist participants in accessing and completing COVID-19 testing sequences in a timely fashion and resolving barriers such as transportation or the possible need to take off work if diagnosed with COVID-19. Level 2: We will provide participants with a self-test kit which can be picked up or mailed to them. We will review how to conduct the test, interpret results, its limitations, and the need to continue prevention guidelines where possible, and provide patient fact sheets as part of the test’s emergency use authorization, in accordance with CDC guidelines [86]. Component D contrasts alternative strategies for addressing structural barriers to testing, since these strategies would be likely to have an antagonistic interaction (each less effective when the other is present) if they were delivered as separate on/off components. Also, we believe some form of enhanced access needs to be part of any multicomponent intervention.

### Criteria for discontinuing or modifying allocated interventions for a given trial participant

Intervention components Core, A, C, and D are individualized, because they are administered individually to the participant by a trained interventionist. The interventionist can discontinue the component upon request or as needed and although the components follow a manual, the component can be modified as needed, consistent with clinical intervention practice. Component B can be discontinued upon participant request. We will attend to social harms and adverse events through the trial.

### Relevant concomitant care and interventions that are permitted or prohibited during the trial

There are no restrictions regarding other types of care or interventions received by participants during the trial.

### Outcomes

The study’s primary outcome is at least one instance of diagnostic COVID-19 testing in the past six weeks (that is, not antibody testing), confirmed with documentary evidence of the result (e.g., a doctor’s note or patient portal electronic health record note [e.g., from a patient portal such as MyChart] that includes the type of test, date of testing, and the result, or a photograph of a self-test result or self-test results).

Secondary outcomes include: result of each COVID test carried out; if diagnosed with or has a positive COVID test result confirmatory testing, as needed (PCR testing is recommended for symptomatic negative and asymptomatic positive persons who carry out a rapid test), contact tracing, self-isolation, health care receipt, and unintended consequences of testing. We will also assess attitudes toward COVID-19 vaccination and receipt of COVID-19 vaccination (the CDC vaccination card will be shown if possible).

### Sample size

We will enroll 448 BLH-FEW. We carried out a power analysis to determine this sample size. For the primary outcome, COVID-19 testing by the final FU, we used PASS 2021 [87] to estimate the sample size needed for individual main effects of intervention components corresponding to odds ratios of 2.0 in logistic regression, given α = 0.05. Assuming participants not receiving or receiving the lowest intensity of each component have a 20% chance of testing by the final FU, a sample size of *n* = 352 (*n* = 22 in each of 16 conditions) provides 80% power to detect an odds ratio of 2.0 (i.e., 20% vs. 33% tested). To account for attrition of up to 20% of enrolled participants, we propose a total sample size of 448 participants (*n* = 28 in each of 16 conditions), ensuring complete data for at least *n* = 22 per condition. Given the proposed sample size, when the main effect of an intervention component on a continuous measure of a secondary outcome or mediator is estimated in a linear model or independent-samples t-test, the sample size provides 80% power to detect a small standardized mean difference (*d* = 0.30). Moderator effects corresponding to an odds ratio of OR = 1 in one subgroup and OR = 4 in another can be detected with 83% power if subgroups sizes are roughly equal. To estimate the size of a mediated effect that can be detected given the proposed sample size, we use the approach described by Vittinghoff and colleagues [88] as implemented in PASS 2021. Given a substantial correlation between an intervention component and a hypothesized mediator (*r* = 0.50), an odds ratio of 1.50 can be detected with > 80% power for the effect of a one-SD increase in a continuous mediator on the COVID-19 testing outcome, controlling for treatment assigned.

### Recruitment

We will use a hybrid recruitment plan with both active outreach and passive strategies to reach BLH-FEW efficiently. Recruitment will focus mainly on the ZIP codes with the lowest rates of vaccination (< 50% fully vaccinated) [89]. The recruitment approach includes: 1) flyers describing the study in English and Spanish. Study staff will use these flyers to directly recruit potential participants using ethnographic street recruitment methods (e.g., recruitment in parks and on the street) [90] and in settings where BLH-FEW are located, but without disrupting work activities; 2) ads placed in the medical research section of free newspapers in English and Spanish, and 3) ads disseminated on social media and Craig’s List. (The recruitment plan does not include peer referral methods to reduce the probability of contamination across intervention conditions.)

### Methods: Assignment of interventions

The sequence of allocations to study conditions will be created by the study statistician (CMC) in the R statistical computing environment [91] by randomly shuffling blocks of allocations (i.e., permuted blocks). There are no stratification factors. Tables containing the allocation sequence will be uploaded to REDCap (Research Electronic Data Capture) by the study statistician. REDCap allows restriction of access to the allocation sequence to users with defined roles, and no staff with direct participant contact will have access to the allocation sequence. REDCap also includes a mechanism for study staff to randomly assign a participant to a condition. Upon random assignment to condition, REDCap stores the assignment made under the participant’s unique identifier and reports the assigned condition to study staff. Staff who enroll and assign participants to conditions will not know potential block sizes and their frequency in the sequence of allocations.

### Blinding (masking)

Staff members and participants are not blinded to intervention condition allocation.

### Methods: Data collection, management, and analysis

#### Data collection methods and data management

REDCap will be used through all study phases*.* REDCap is a secure web-based application for building and managing online surveys and databases [92].

**Fig. 3:**
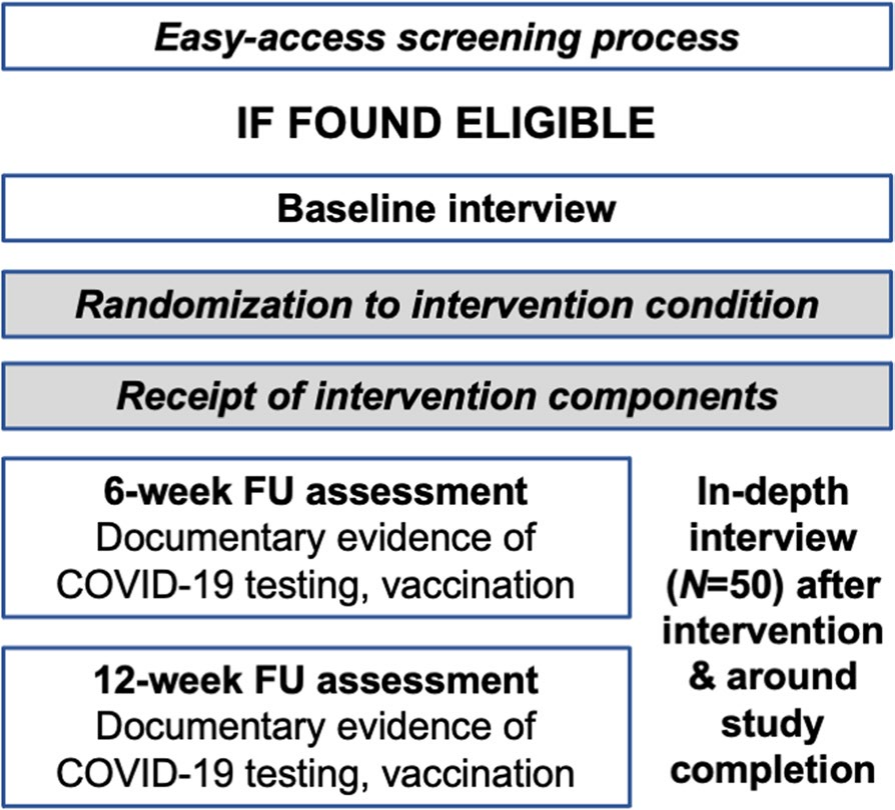
Sequence of study activities

Participants are first screened for eligibility. Potential participants will provide verbal informed consent following an IRB-approved script and participate in a brief (< 15 min) structured screening interview using the Computer-Assisted Personal Interview (CAPI) format in REDCap to determine eligibility. Those found eligible will provide locator information. Screening can take place in recruitment venues, the NYU study field site, or virtually (phone or Voice over Internet Protocol such as Zoom; Fig. 3). Consent will be obtained by trained NYU research staff personnel.

Participants can enroll into the study in-person or virtually. Virtual enrollment is useful if participants are concerned about COVID-19 risk or have recently been exposed to COVID.

When enrolled in-person participants will provide electronic signed informed consent in REDCap (using the “eConsent” feature). They will receive a copy of the consent form for their records. Then they will complete a more detailed locator form to facilitate longitudinal FU, and participate in a structured BL interview using CAPI and the Audio Computer-Assisted Self-Interview (ACASI) format programmed in REDCap.

When enrolling virtually, participants will provide verbal informed consent following an IRB-approved script and a copy of the consent form will be mailed to them. They will then complete the BL interview using ACASI or CAPI (depending on their preference).

After completing the BL, participants will be randomly assigned to an intervention condition using a randomization table in REDCap. Then, they have the opportunity to engage in the core session, or to schedule it for the next 1–2 weeks. Typically, the BL and core session will be carried out on the same day. Intervention components will be administered within the next 1–2 weeks. Components are brief or carried out mainly independently, thus components will generally be implemented in a single meeting. FU assessments will be carried out 6- and 12-weeks post-BL in ACASI and/or CAPI in the REDCap platform.

The BL will last approximately 40–60 min and FU interviews approximately 30–40 min. BL interviews will assess the lifetime and recent period, and the 6- and 12-week FUs will assess the period since the last interview. The measures to be used are drawn primarily from the NIH RADx-Up initiative Common Data Elements (CDEs) [93]. These are reliable and valid measures designed for under-represented populations such as BLH-FEW and measures have been used in hundreds of previous RADx-UP studies.

### Plans to promote participant retention and complete FU

We are leaders in the development of tracking and retention strategies with populations such as including BLH-FEW. Successful tracking and retention over time is a multifaceted effort requiring simultaneous strategies at the management, staff, participant, and compensation levels.

Retention to intervention components and assessments is monitored daily.

Regarding aspects of the staff and project, the conduct and training of staff and ethos of the study can communicate trustworthiness and thereby facilitate engagement. In the proposed study, staff will be trained to respect participants’ decisions about their engagement with the study without judgment or pressure. The intervention activities similarly take an autonomy-supportive approach. Participants can decline COVID-19 testing or any other study activity and remain in the study. Compensation levels must be perceived as fair and appropriate for the amount of time participants will spend engaging in activities. Further, ClinCard allows us to provide compensation immediately after study activities are completed. Reducing undue burden (e.g., assessments less than one hour in length) and making participation as convenient as possible (e.g., weekend and evening hours, virtual participation, rescheduling as necessary) can further facilitate retention.

A locator form is a crucial component of the tracking and retention strategy. At the time of enrollment and after providing signed informed consent, the participant will complete a detailed locator form, which is housed in the REDCap database. The project staff member will solicit the names and contact information of at least three individuals who will know how to reach the participant in the future. Participants may provide the names of case managers and other professionals, as well as personal contacts. We will obtain email addresses, cell phone numbers, Facebook IDs, and other social media IDs, if participants are comfortable being contacted at these virtual locations. The project will maintain a Facebook page.

Locator information will be reviewed with participants at study contacts and updated as needed. Because attrition is highest in the earliest phases of a study, retention efforts will begin shortly after enrollment. We will maintain contact via mail: a thank-you card will be sent to the participant after the BL. This is a useful way to maintain rapport and returned mail will help identify participants at risk of becoming lost to FU. All cards, recruitment, and retention materials will be approved by the IRB.

Tracking participants begins with a direct approach: contacting the participant by phone, text, email, social media, and/or mail. If direct tracking is not successful, we will begin more intensive outreach efforts. The first step will be to contact network members listed on the locator form. If network-based tracking methods are unsuccessful, we will initiate systems-level tracking. At enrollment, participants will have been asked to list agencies with which they have contact, and we will send a letter or flyer to each of these agencies requesting that they forward the enclosed letter to the participant’s most current address. Additionally, we will use free systems-level tracking methods commonly used to locate participants in longitudinal studies. These include searching web-based phone. In some cases, we will attempt place-based tracking, canvassing locations where the participant reports often spending time.

Participants who decline to engage in intervention activities will still be scheduled to complete FU assessments.

### Statistical methods

Intent-to-treat analysis will be our primary analytic approach and exploratory analyses will examine complier average effects of intervention components [94, 95]. Approaches to missing data will include full information maximum likelihood estimation [96] and multiple imputation [97]. In sensitivity analysis, missing data will be treated as failure to achieve the desired outcome. If data are missing not at random (MNAR), we will employ sensitivity analysis, using selection [98] or pattern mixture [99, 100] models.

### Aim 1

Identify which of four components or interactions between components contribute meaningfully to improvement in the primary outcome, COVID-19 testing with documentary evidence, and from these results, optimize a multicomponent intervention**.**

The primary outcome for Aim 1 is COVID-19 testing by the final FU point (12-weeks post-BL). Logistic regression will be used to estimate effects of components on the odds of COVID-19 testing. Intervention components will be effect-coded to estimate main effects, two-way, three-way, and four-way interactions of all four components (see Eq. 1). The coefficient for an effect-coded main effect term (e.g., *b*_*1*_), multiplied by two and exponentiated, will estimate the effect of the component (e.g., Component A) on the odds of testing. Similarly, the coefficient for an effect-coded interaction term, multiplied by two and exponentiated, will estimate interaction effects between or among components on the odds of testing. Similar logistic regression analyses will estimate effects of components on secondary outcomes.

1 $$\log\;\left(\frac{{\mathrm\pi}_{\mathrm i}}{1-{\mathrm\pi}_{\mathrm i}}\right)=b_0+b_1X_A+b_2X_B+b_3X_c+b_4+X_D+b_5X_{A\ast B}+b_6X_{A\ast C}+b_7X_{A\ast D}+b_8X_{B\ast C}+b_9X_{B\ast D}+b_{10}X_{C\ast D}+b_{11}X_{A\ast B\ast C}+b_{12}X_{A\ast B\ast D}+b_{13}X_{A\ast C\ast D}+b_{14}X_{B\ast C\ast D}+b_{15}X_{A\ast B\ast C\ast D}$$

Intervention optimization. Based on main and interactive effects estimated in Aim 1, we will use a decision-making process to select the most effective combination of component levels, eliminating ineffective or poorly performing components. The decision-making process will be led by the study leadership team (the Principal Investigator and Co-Investigators) and using procedures outlined in Collins [48]. To identify important component main effects, we consider both statistical significance at p < 0.05 as well as the probability the more effective component level multiplies the odds of testing by at least OR = 1.2. This effect size threshold is based on the idea that several components with an effect as large or larger would comprise a potent multicomponent intervention. We will use a Bayesian generalized linear model with non-informative priors to calculate the probability of OR ≥ 1.2 from the posterior distribution. Component main effects that are statistically significant (p < 0.05) and have a probability > 0.5 of OR ≥ 1.2 will be considered important and placed into a screened-in set. Membership in the screened-in set will be reconsidered in light of any important interactions. Interactions that are statistically significant (p < 0.05) and have a probability > 0.5 of OR ≥ 1.2 will be considered important. For example, a component level that does not meet criteria for an important main effect could be included if it enhances the effectiveness of another component. Component levels that make up the optimized intervention are comprised of the higher levels from the screened-in set and the lower levels from the screened-out set. 

### Aim 2

Identify mediators and moderators of the efficacy of each intervention component. To examine potential mediating mechanisms, analysis for Aim 2 will use the potential outcomes framework [101–103]. This framework highlights assumptions needed to identify direct and indirect effects of interest: no unmeasured cofounders of the exposure (an intervention component) and outcome (COVID-19 testing) relation; no unmeasured confounders of the mediator and outcome relation; no unmeasured confounders of the exposure and mediator relation; and no measured or unmeasured confounders of the mediator and outcome relation affected by exposure. Since intervention components are randomly assigned, the key issue for the proposed study is addressing confounding of the relation between mediators and outcomes. Mediators measured at BL will be included as confounders of the relation between FU mediators and COVID-19 testing by the FU. Because unmeasured confounding of relations between mediators and outcomes may remain despite attempts to measure and include known confounders in the models, sensitivity analysis will be undertaken to determine how the size of the correlation between error for the mediator model and error for the outcome model impacts inferences for direct and indirect effects. The total natural indirect effect (TNIE) and pure natural direct effect (PNDE) of each component will be estimated using the *mediation* R package [104]. The TNIE compares the outcome when subjects are exposed (e.g., receive a component), and the mediator varies as it would naturally under exposure, versus the outcome when subjects are exposed but the mediator varies as it would naturally in the absence of exposure (i.e., component not received). In other words, the difference estimated by the TNIE compares the expected outcome when the intervention has its natural impact on the mediator versus the expected outcome when the action of the mediator is blocked. The PDNE compares participants at different levels of a component (e.g., on vs. off) when a mediator is blocked.

Potential moderator effects will be examined by adding interaction terms to the model described for **Aim 1**. We will include sociodemographic characteristics (e.g., age) and occupation as covariates and explore the interactions of these variables with intervention components. When interaction effects are detected, we will estimate the simple main effects of the intervention component across levels of the moderator variable (e.g., MI counseling effects on testing for younger vs. older participants). Identified moderators will inform future adaptive interventions [105].

### Methods: Monitoring

#### Data monitoring

This is a low-risk study and as such there is no Data and Safety Monitoring Board for this study.

### Harms

Participant safety will be monitored at FU assessments by queries regarding potential adverse events as well as social harms related to study participation in domains such as occupation, health care, and housing.

### Ethics and dissemination

#### Research ethics approval

The study is approved by the University Committee on Activities Involving Human Subjects at New York University (FWA#00,006,386).

### Protocol amendments

Protocol amendments will be approved by the University Committee on Activities Involving Human Subjects prior to implementation. The Program Official at the National Institutes of Minority Health and Health Disparities will approve any substantive changes to the protocol.

### Consent

Informed consent will be obtained by trained research study staff members. We will obtain verbal informed consent following an IRB-approved script for the screening interview. We will obtain signed informed consent for enrollment if the participant is enrolled in person or verbal informed consent following an IRB-approved script if enrolled virtually (e.g., because of COVID concerns). Participants will receive a copy of the enrollment Informed Consent Form regardless of the type of consent provided (signed vs. verbal consent).

### Confidentiality

All participants will receive a Participant Identification Number (PIN) that will be used for all interviews, consent forms, materials, transcripts, and intervention materials. No other information that would disclose the participant’s identity will be found on any interview or form. Study staff do not collect paper forms; all materials are located in the secure REDCap database. Participants provide signed consent electronically and the signature is recoded in REDCap. Staff receives training about confidentiality. Participants will be provided a paper copy of the consent form that includes contact information for the research team Principal Investigator and the Institutional Review Board as appropriate. Participants can use this contact information to report adverse events or unanticipated problems.

### Access to data

Access to data will be restricted to the research team at NYU. Study members outside NYU may have access to the data after completion of a data sharing agreement. A limited data set (without identifiers) will be provided to the RADx-UP data coordinating center. This is a condition of our Notice of Award. Participants will be informed that data will be provided to RADx-UP during the informed consent process.

### Declaration of interests

The Principal Investigator or Co-Investigators do not have any financial and other competing interests.

### Dissemination policy

We will disseminate study findings in a timely fashion through presentations at scientific meetings, scientific publications, mainstream and scientific media such as newsletters and professional publications, press releases, social media, and other venues.

One goal of the proposed study is to create an implementation strategy manual to guide implementation of the new optimized intervention in community-based organizations. The manual will detail training requirements and recommendations for implementation in each type of setting, for rapid scale-up and maximum public health benefit. In collaboration with NMIC, we will disseminate the implementation strategy manual and intervention manual that describes the optimized intervention.

We will publicize the results of the study to community-based organizations, funders, public health officials, scientists, clinicians, policy makers, research participants, and the general public through the following venues: Publications in scientific journal, presentations at scientific and clinical conferences, presentations at scientific grand rounds at local, national, and international institutions, development of symposia to present study findings, articles in scientific and lay newsletters or blogs tailored to participants, clinicians, and other stakeholders, press releases describing studies or individual papers written by the NYU Silver School of Social Work communications team, interviews with local media, and content presented on social media managed by Silver and NYU at large (Facebook, Twitter) and NMIC.

The study will participate in activities organized by the RADx-UP Coordinating and Data Collection Center (CDCC), including participating in regular meetings, collaborating with RADx-UP working groups, and data sharing and dissemination activities. The study will share data collection instruments (e.g., survey items, code books), other research products (e.g., informed consent forms, data collection forms) and data to facilitate data harmonization efforts across RADx-UP project sites.

We will disseminate findings on social media. NYU Silver's Communications Department disseminates research findings and news via the School's website, social media platforms (Twitter, Facebook, LinkedIn, Instagram), email, and newsletters. The Department also collaborates with NYU's central departments of Public Affairs, Government Affairs, and Community Engagement to disseminate findings to relevant reporters and media outlets, policy makers, and stakeholders. In addition, the Department promotes lectures, webinars, and publications that further extend and amplify research content. NMIC maintains an active social media presence as well.

The study is registered with clinicaltrials.gov. The PI will ensure that the proposed study is registered on clinicaltrials.gov as outlined in the NIH policy (NOTOD-16–149). With this method, researchers and potential participants will be able to contact us. The PI will complete this registration no later than 21 calendar days after the enrollment of the first participant and will maintain the record over the course of the study. The PI will post study results on ClinicalTrials.gov within one year after the study’s primary completion date.

We will make study results available to participants. Community engagement has become an important ethical requirement for research involving human subjects, particularly populations that experience challenges to health and wellbeing. One step towards engaging the community is making study results available to study participants. Study findings will be placed on a project page on the NYU Silver website and NMIC’s website at the conclusion of the study. We will also hold an in-person meeting at the conclusion of the study to review findings with participants and gather their reactions and input.

## Discussion

This optimization trial is designed to yield an effective, affordable, and efficient behavioral intervention that can be rapidly scaled in community settings. Further, it will advance the literature on intervention approaches for social inequities such as those evident in the COVID-19 pandemic.

## Supplementary Information

**Additional file 1.**

## Data Availability

The datasets used and/or analysed during the current study are available from the corresponding author on reasonable request.

## Abbreviations

ACASI: Audio computer-assisted self-interview
BE: Behavioral economics
BL: Baseline
BLH: Black and Latino/Hispanic
CAB: Community advisory board
CDC: Centers for Disease Control and Prevention
CDCC: Coordinating and Data Collection Center
CAPI: Computer-assisted personal interview
CDE: Common data elements
FEW: Frontline essential workers
FU: Follow-up
IIT-ICM: Intervention Innovations Team integrated conceptual model
MI: Motivational interviewing
MNAR: Missing not at random
MOST: Multiphase optimization strategy
NAAT: Nucleic acid amplification
NMIC: Northern Manhattan Improvement Corporation
NYU: New York University
PDI: Peer-driven intervention
PNDE: Pure natural direct effect
QQ: Quiz questions
RCT: Randomized controlled trial
REDCap: Research Electronic Data Capture
TM: Text messaging
TNIE: Total natural indirect effect
